# Inhibition of the Sterol Regulatory Element Binding Protein SREBF-1 Overcomes Docetaxel Resistance in Advanced Prostate Cancer

**DOI:** 10.1016/j.ajpath.2024.07.019

**Published:** 2024-08-19

**Authors:** Maximilian P. Brandt, Olesya Vakhrusheva, Hubert Hackl, Tamas Daher, Katrin Tagscherer, Wilfried Roth, Igor Tsaur, Florian Handle, Andrea Eigentler, Zoran Culig, Christian Thomas, Holger H.H. Erb, Axel Haferkamp, Eva Jüngel, Martin Puhr

**Affiliations:** ∗ Department of Urology and Pediatric Urology, Mainz University Medical Center, Mainz, Germany; † Department of Urology, University of Tuebingen, Tuebingen, Germany; ‡ Institute of Bioinformatics, Biocenter, Medical University of Innsbruck, Innsbruck, Austria; § Institute of Pathology, Mainz University Medical Center, Mainz, Germany; ¶ Optipath, Ambulatory Health Care Center for Pathology Frankfurt, Frankfurt, Germany; ‖ Department of Urology, Medical University of Innsbruck, Innsbruck, Austria; ∗∗ Institute of Pathology, Neuropathology and Molecular Pathology, Medical University of Innsbruck, Innsbruck, Austria; †† Department of Urology, Technische Universität Dresden, Dresden, Germany

## Abstract

Resistance to antiandrogens and chemotherapy (Cx) limits therapeutic options for patients with metastatic hormone-sensitive (mHSPC) and metastatic castration-resistant (mCRPC) prostate cancer. In this context, up-regulation of the glucocorticoid receptor is identified as a potential bypass mechanism in mCRPC. A combination of docetaxel and mifepristone (Doc + RU-486), an inhibitor of the glucocorticoid receptor, re-sensitizes docetaxel-resistant cell models to Cx. This study was designed to elucidate the molecular mechanisms responsible for this phenomenon. RNA sequencing was performed in docetaxel-resistant prostate cancer cell models after Doc + RU-486 treatment with consecutive functional assays. Expression of selected proteins was verified in prostatic tissue from prostate cancer patients with progressive disease. Treatment with Doc + RU-486 significantly reduced cancer cell viability, and RNA sequencing revealed sterol regulatory element of binding transcription factor 1 (SREBF-1), a transcription factor of cholesterol and lipid biosynthesis, as a significantly down-regulated target. Functional assays confirmed that SREBF-1 down-regulation is partially responsible for this observation. In concordance, SREBF-1 knockdown and pharmacologic sterol regulatory element binding protein inhibition, together with other key enzymes in the cholesterol pathway, showed similar results. Furthermore, SREBF-1 expression is significantly elevated in advanced prostate cancer tissues, showing its potential involvement in tumor progression and emerging therapy resistance. Therefore, specific inhibition of cholesterol and lipid biosynthesis might also target Cx-resistant cancer cells and represents a potential additive future therapeutic option to improve mCRPC therapy.

Over the past decade, treatment options for patients with metastatic hormone-sensitive prostate cancer (mHSPC) and metastatic castration-resistant prostate cancer (mCRPC) have dramatically increased with improved disease control by prolonging progression-free survival and overall survival.^1^, ^2^, ^3^, ^4^, ^5^, ^6^ Docetaxel, one of the most established chemotherapy regimens for prostate cancer (PCa), is approved for patients with mHSPC and mCRPC and remains a cornerstone of the treatment algorithm for patients with PCa.^7^^,^^8^ Combining docetaxel with second-generation hormonal therapy, such as abiraterone acetate or the androgen receptor (AR) inhibitor darolutamide, is a novel treatment approach. Recent data from the phase 3 PEACE-1 (A Phase III Study for Patients with Metastatic Hormone-naïve Prostate Cancer) and ARASENS (ODM-201 in Addition to Standard ADT and Docetaxel in Metastatic Castration Sensitive Prostate Cancer) trials have reported improved progression-free survival and improved overall survival.^9^^,^^10^ However, the efficacy of chemotherapy is restricted by the development of therapy resistance, which represents a significant limitation in clinical practice. Molecular changes that correlate with acquired therapy resistance to taxane medication include tubulin alterations, overexpression of ERG, increased expression of the transporter ABCB1, and up-regulation of the glucocorticoid (GR) and AR networks.^11^, ^12^, ^13^ Elevated GR expression is a potential driver of therapy resistance, and it accelerates PCa tumor progression.^14^, ^15^, ^16^ In addition, PCa cells show increased intratumoral steroidal, cholesterol, and lipid synthesis, which contributes to the decreased efficacy of antiandrogens and chemotherapy.^17^^,^^18^

Because androgen synthesis largely depends on functional cholesterol synthesis, inhibiting enzymes of the cholesterol pathway represents a meaningful target in PCa. For instance, abiraterone acetate is highly effective in patients with mHSPC and mCRPC because it inhibits cytochrome P450 17A1, a central enzyme in the steroid synthesis pathway.^19^

Sterol regulatory element binding proteins (SREBPs) are key regulators of cholesterol and lipid biosynthesis, and increased SREBP expression is associated with accelerated tumor progression in many malignancies, including PCa.^20^, ^21^, ^22^, ^23^ Importantly, SREBPs can directly interact with DNA binding sites and regulate the expression of other key enzymes in the mevalonate pathway, such as 3-hydroxy-3-methylglutaryl-coenzyme-A–reductase (HMGCoA-R), 3-hydroxy-3-methylglutaryl-coenzyme-A–synthase, or squalene epoxidase, as well as lipid metabolism.^24^ Furthermore, elevated SREBP expression is linked to therapy resistance in other malignancies such as melanoma. In addition, SREBPs are involved in AR regulation, and their increased expression is associated with unfavorable PCa survival.^25^, ^26^, ^27^

Inhibition of GR with mifepristone (RU-486) in combination with docetaxel induces sensitivity to docetaxel; however, the mechanisms responsible for this finding are unknown. To elaborate on the observations of reduced cell viability and elevated apoptosis under combination treatment, a gene analysis approach was chosen to identify the significantly altered genes potentially responsible for these findings. Docetaxel treatment combined with RU-486 significantly reduced *SREBF1* expression and impaired cholesterol and lipid biosynthesis. Moreover, sterol regulatory element of binding transcription factor 1 (SREBF-1) was significantly elevated in tissues from patients with advanced PCa, indicating the strong involvement of SREBPs in PCa progression and therapy resistance. In summary, based on the preclinical results, inhibition of cholesterol synthesis via SREBF-1 may represent a promising tool to improve the efficacy of docetaxel-based chemotherapy in mCRPC.

## Materials and Methods

### Cell Culture

PC3, DU145, and CWR22Rv1 cells were obtained from the ATCC (Manassas, VA). Docetaxel-resistant (DR) CWR22Rv1 (CWR22Rv1-DR) cells were kindly gifted by Professor William Watson (University College Dublin, Dublin, Ireland). DR PC3 and DU145 (PC3-DR and DU145-DR) cells were generated after long-term treatment with increasing doses of docetaxel up to a final concentration of 12.5 nmol/L and cultured as previously described.^14^^,^^28^ All cell lines were cultured in RPMI 1640 medium supplemented with 10% fetal calf serum (Biowest, Riverside, MO), 1% penicillin/streptomycin (Szabo-Scandic, Vienna, Austria), and GlutaMAX 1X (Thermo Fisher Scientific, Vienna, Austria) in the presence or absence of 12.5 nmol/L docetaxel (Sigma-Aldrich, Vienna, Austria). The authenticity of all cell lines was validated by using short tandem repeat profiling.

### RNA Isolation from Cell Lines

Total RNA from cell lines was isolated using the Blirt EXTRACTME TOTAL RNA Kit (LabConsulting, Vienna, Austria) according to the manufacturer’s instructions. RNA yield and quality were determined by using a NanoDrop 2000 system (Thermo Fisher Scientific).

### RNA Isolation from Prostate Tissue

mRNA isolated from 40 representative radical prostatectomy patients was analyzed for *SREBF1* expression. Patients were selected from the Biobank of the Department of Urology of the Medical University of Innsbruck (Table 1). The use of archived materials was approved by the Ethics Committee of the Medical University of Innsbruck (EV 1072/2018). Written consent was obtained from all patients and documented in the database of the University Hospital Innsbruck in agreement with statutory provisions. Tissue handling and RNA extraction have been previously described in detail.^14^

**Table 1:** Patient Characteristics and Descriptive Histology at Time of RPE

Patients (*n* = 40)	Mean	Range
Age, y	61.35	47–74
PSA diagnosis, ng/mL	6.6	1.8–44.24
Free PSA, %	14.25	0–28.9
Prostate volume, g	40.97	18–70
GSC at time of RPE	*n*	%
GSC 5–6	6	15
GSC 7	29	73
GSC 8–10	5	13
pT stage at time of RPE	*n*	%
pT2a	2	5
pT2b	0	0
pT2c	22	55
pT3a	11	28
pT3b	4	10
pT4	1	3
Tumor relapse	No	Yes
Patients	35	5
Time to relapse, months	32.88	4–64.4

GSC, Gleason score; PSA, prostate-specific antigen; RPE, radical prostatectomy.

### cDNA Synthesis and Quantitative RT-PCR

cDNA synthesis was performed by using a Luna Script RT Super Mix Kit (New England Biolabs, Ipswich, MA). Quantitative RT-PCR was performed on an ABI PRISM 7500-FAST system (Thermo Fisher Scientific) using a Luna Universal Probe qPCR Master Mix (New England Biolabs) according to the manufacturer’s protocol. TBP, HPRT1, and HMBS were used as housekeeping genes to normalize data. The custom primer and probe sequences were as follows: TBP (forward: 5ʹ-CACGAACCACGGCACTGATT-3ʹ; reverse: 5ʹ-TTTTC-TGCTGCCAGTCTGGAC-3ʹ; probe: 5ʹ-FAM-TCTTCACT-CTTGGCTCCTGTGCACA-TAMRA-3ʹ), HPRT1 (forward: 5ʹ-GCTTTCCTTGGTCAGGCAGTA-3ʹ; reverse: 5ʹ-GTCT-GGCTTATATCCAACACTTCGT-3ʹ; probe: 5ʹ-FAM-TC-AAGGTCGCAAGCTTGCTGGTGAAAAGGA-TAMRA-3ʹ),HMBS (TaqMan Gene Expression Assay from Thermo Fisher Scientific; Hs00609297_m1), NR3C1 (Hs00353740_m1), and SREBF-1 (Hs01088679_g1). TaqMan gene expression assays (Thermo Fisher Scientific) were used according to the manufacturer’s protocol.

### Western Blot Analysis

Western blot analyses were performed as previously described to assess the expression of proteins that are directly involved in proliferation and apoptosis.^29^ Cells were lysed in lithium dodecyl sulfate sample buffer, and, after protein quantification according to the Bradford method, 50 μg or 75 μg total protein was separated on 4% to 12% Bis-Tris gels (Expedeon, San Diego, CA) or 4% to 12% polyacrylamide gels and transferred onto 0.2 μm nitrocellulose membranes (GE Healthcare, Chicago, IL). Membranes were blocked, incubated with 5% bovine serum albumin in Tris-buffered saline overnight, and then incubated with the secondary antibody. The following antibodies were used: anti-GAPDH (1:50.000; MAB374; Millipore, Vienna, Austria), anti-Vinculin (sc-25336, 1:500: Santa Cruz Biotechnology, Dallas, TX), anti–lamin A (ab264322, 1:2000; Abcam, Cambridge, United Kingdom), anti–α-tubulin (sc-23948, 1:500; Santa Cruz Biotechnology), anti-GR rabbit mAb (D6H2L) (#12041, 1:500; Cell Signaling Technology, Danvers, MA), anti–p-AKT (1:2000; Cell Signaling Technology S473 (#4060, D-9E), anti-AKT (9272S, 1:2000; Cell Signaling Technology), anti-BCL2 (2870S, 1:2000; Cell Signaling Technology, 50E3), anti–MCL-1 (SC-819, 1:2000; Santa Cruz), anti–c-PARP (G7341, 1:500; Sigma, Taufkirchen, Germany), anti–SREBF-1 (SC-17755, 1:500, 1:1000 and 1:2000, Santa Cruz Biotechnology), and anti–HMGCoA-R (AMAb90618; Atlas Antibodies, Bromma, Sweden).

### Cell Viability

A total of 2.5 × 10³ PCa cells per well were seeded into 96-well plates. After 24 hours of cell attachment, the medium was removed, and treatment with the indicated drugs was initiated for 5 days. Briefly, treatment was performed with or without 12.5 nmol/L docetaxel, 3 μmol/L and 10 μmol/L RU-486, 6.25 μg fatostatin (an SREBP inhibitor), and 0.5 μmol/L (PC3-DR) or 6.25 μmol/L (DU145-DR) simvastatin (an inhibitor of HMGCoA-R). Dimethyl sulfoxide (DMSO) was used as a vehicle control in untreated control cells. On day 5 of treatment with docetaxel, RU486, or their combination, cell viability was determined. In all other experiments with the pharmacologic inhibitors fatostatin and simvastatin, cell viability was determined after 72 hours with increasing doses up to 25 μmol/L and 10 μmol/L, respectively. The absorbance at 570 nm was determined for each well by using a multimode microplate reader (Spark 10 mol/L; Tecan, Crailsheim, Germany). Results are expressed as mean cell numbers after subtracting the background absorbance and offsetting it with a standard curve. To illustrate dose-response kinetics, the mean cell number after 24 hours of incubation was set to 100%. Each experiment was performed with a minimum of three biological replicates.

### Inhibitory Concentration of Docetaxel on Cell Viability

A total of 2.5 × 10³ PC3-DR, DU145-DR, and CWR22Rv1-DR cells per well were seeded into 96-well plates and incubated for 24 hours for cell attachment. After 24 hours, the medium was removed, and the cells were treated with a decreasing docetaxel concentration as listed (ie, 12.5 μmol/L, 10 μmol/L, 7.5 μmol/L, 5 μmol/L, 2.5 μmol/L, 1 μmol/L, 0.75 μmol/L) and control (no treatment) in each row of the 96-well plate combined with 1 μmol/L, 3 μmol/L, and 10 μmol/L RU-486. Cell viability was assessed after incubation for 5 days using cell viability assays, as described in the previous paragraph.

### Cell Death Analysis

Cell death was assessed by using the propidium iodide assay. PC3-DR, DU145-DR, and CWR22Rv1-DR cells were seeded in petri dishes. Each petri dish was treated with or without 12.5 nmol/L docetaxel in combination with 3 μmol/L RU-486 and 10 μmol/L RU-486 DMSO (vehicle). Because of the observed intense effect on cell viability after 5 days, cell death analysis was performed after 72 hours. The percentage of apoptotic cells (sub-G1) was determined by using the FACSCalibur flow cytometer (Becton Dickinson, Heidelberg, Germany).

### siRNA Transfection

For siRNA transfection, ON-TARGETplus technology with an siRNA SMARTpool for SREBF-1 inhibition was used according to the manufacturer’s instructions (Dharmacon, Chicago, IL). A nontargeting siRNA pool was used as a negative control. Briefly, siRNA transfection was performed by using Lipofectamine 2000 reagent (Invitrogen, Carlsbad, CA) according to the manufacturer’s protocol. All cell lines were transfected with 50 nmol/L SMARTpool siRNA against *SREBF1* with either a nontargeting control or no siRNA. To ensure prolonged knockdown of the target proteins for 6 days, all cell lines were re-transfected with the same concentration of the respective siRNA on day 3.

### RNA Sequencing and Bioinformatics

The PC3-DR and DU145-DR cell lines were used for RNA sequencing (RNA-seq). All treatments for each cell line were used for the RNA-seq analysis. The analysis was performed for 48 hours; 5 × 10^6^ PC3-DR and DU145-DR cells (5 × 10^6^ cells) were seeded in petri dishes. Specific treatments were performed 24 hours after seeding. The treatment groups were as follows: Group 1, vehicle CTRL cells treated with DMSO; Group 2, cells were treated with docetaxel 12.5 nmol/L; Group 3, cells were treated with RU-486 10 μmol/L; and Group 4, cells were treated with docetaxel 12.5 nmol/L and RU-486 10 μmol/L. All experiments were performed in triplicate.

RNA was isolated as described in the previous paragraph. Sequencing was performed on an Illumina NextSeq sequencing device by a service provider (StarSEQ, Mainz, Germany) according to standard protocols. The RNA-seq files were trimmed for adaptor sequence removal, quality was controlled using Trimmomatic (0.36), and the reads were trimmed to 148 bases. The sequencing quality was assessed by using FastQC version 0.11.8. Reads were mapped using the STAR splice-aware aligner (version 2.6.0c) on the human genome version hg38 (UCSC) with RefGene annotation and index with 200 bases splice junction overhang. HTSeq was used to quantify the raw gene counts. Differentially expressed genes and normalization (regularized log expression) were analyzed by using the R package DESeq2. Differentially expressed genes between each treatment group and control treatment (DMSO) were identified based on a negative binomial distribution using DESeq2.

*P* values were adjusted for multiple testing based on the false discovery rate according to the Benjamini-Hochberg method. Genes with more than twofold change and adjusted *P* value (false discovery rate) < 0.1 were considered significantly differentially expressed. The *z* scores from normalized expression (log2 of normalized counts adding a pseudocount of 1) were visualized as heatmaps using Genesis version 1.8.1 for genes significantly differentially expressed between docetaxel + RU-486 versus DMSO. Log2-fold changes of the top 30 up-regulated and 30 down-regulated genes in both cell lines between docetaxel + RU-486 versus DMSO were indicated as a heatmap (ranked by average log2-fold changes of the two cell lines). Commonly up-regulated and down-regulated genes of docetaxel + RU-486 versus DMSO between both cell lines PC3-DR and DU145-DR were identified by using a Venn diagram. The data were deposited in the National Center for Biotechnology Information Gene Expression Omnibus (*https://www.ncbi.nlm.nih.gov/geo*; accession number GSE233647).

### Public Data Sets and TCGA Data Sets

For the integrated analysis of SREBF-1 mRNA expression, the TCGA-PRAD,^30^ GSE21034,^31^ GSE35988,^32^ and GSE62872^33^ (*https://www.ncbi.nlm.nih.gov/geo*) data sets were merged and analyzed by using the R package limma (version 3.44.3). Differential gene expression analysis was performed by using the treatment pipeline with the project as a blocking variable. For visualization, the batch effect between projects was removed by using a built-in function in the limma. The gene set activity scores for the AR- and GR-selective target gene sets were individually calculated using GSVA for TCGA-PRAD (*n* = 547 samples) and SU2C^34^ (*n* = 266 samples) data sets. The Pearson correlation coefficient was used to calculate the correlation between SREBF-1 log2 mRNA expression and gene set activity scores. As encoded in the figure legends, all differences highlighted by asterisks are statistically significant (∗*P* < 0.05, ∗∗*P* < 0.01, ∗∗∗*P* < 0.001). Data are presented as the means ± SE or SEM unless otherwise specified.

### Patient Material for Immunohistochemistry

Paraffin-embedded tissue samples from 72 patients with advanced PCa who had obstructive lower urinary tract symptoms and underwent transurethral resection of the prostate were provided by the Tissue Biobank of the University Medical Center Mainz (Table 1). Eighty-four patients with benign prostatic hyperplasia served as the controls. Patients with obstructive PCa were considered hormone sensitive if they were under hormonal suppression/deprivation treatment that consisted of orchiectomy or androgen deprivation therapy (luteinizing hormone or first-generation antiandrogen) and did not show any increase in prostate-specific antigen levels. Patients who had received multiple systemic treatment lines at the time of surgery for obstructive voiding symptoms and therefore had already passed the state of hormone sensitivity to castration resistance were considered mCRPC. Patient material acquisition was approved by the regulations of the Tissue Biobank University Medical Center (Mainz, Germany) after approval of the Regional Ethics Committee, Rhineland-Palatinate (Study No. 2020-15463) as well as the Ethics Committee of the Medical University of Innsbruck.^14^

A tissue microarray containing cores derived from transurethrally resected prostatic tissue with histologically confirmed PCa (defined as the primary tumor) and benign prostatic tissue from control subjects was established. After antigen retrieval at pH 9.0, the tissue microarray was stained with the respective antibody SREBF-1 (1:2000, #66875-1-Ig; Proteintech, Rosemont, IL) for 30 minutes and the EnVision FLEX HRP/DAB Kit (K 8010; Dako, Agilent, Santa Clara, CA) using an automated staining system (Autostainer 480S-2D; Epredia, Kalamazoo, MI). After digitalization using the NanoZoomer Slide Scanner (Hamamatsu Photonics, Hamamatsu, Japan), digital analysis of the tissue microarray was performed by using QuPath software version 0.1.1,^35^ including classification steps and training of the program to identify and distinguish between epithelial and stromal tumor cell populations. After successful training, the staining intensity in the cytoplasmic, nuclear, cytoplasmic, and nuclear compartments within the epithelial cells was evaluated. The SREBF-1 expression intensity was manually assigned as negative (0), weak (1+), moderate (2+), or strong (3+). The histochemical score, a measure of nuclear immunoreactivity, was calculated for each core, depending on the extent and intensity of staining; scores ranged from 0 to 300.

### Statistical Analysis

GraphPad Prism 9 (GraphPad Software, La Jolla, CA) was used for statistical analyses. Gaussian distribution was determined by using Kolmogorov-Smirnov testing and D’Agostino and Pearson omnibus normality tests. Differences between treatment groups were analyzed by using *t*-tests or *U*-tests depending on Gaussian distribution. Comparison of multiple treatment groups was made by using one-way analysis of variance and pairwise post hoc tests corrected for multiple testing using Bonferroni or Dunn’s multiple comparison test methods depending on Gaussian distribution.

## Results

### Administration of RU-486 in Combination with Docetaxel Results in Elevated Apoptosis in DR Cells

**Figure 1:**
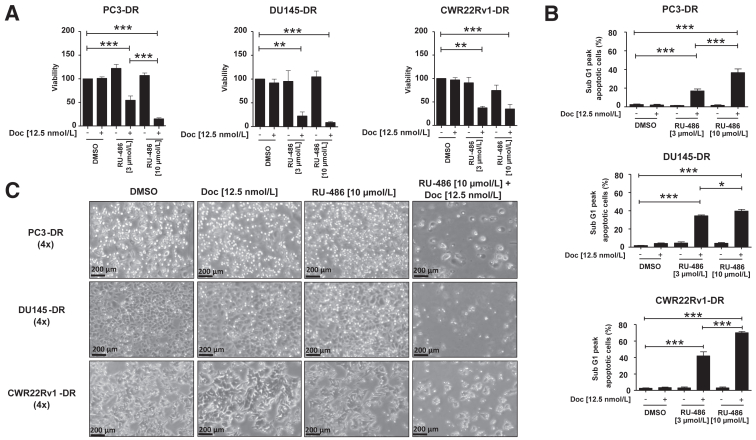
**A:** Significantly reduced cell viability in the docetaxel (Doc)-resistant cell models PC3-DR, DU145-DR, and CWR22Rv1-DR after 3 days of treatment with RU-486 (10 μmol/L) in combination with Doc (12.5 nmol/L) compared with single treatment of Doc (12.5 nmol/L) and RU-486 (10 μmol/L). **B:** Flow cytometry analysis shows increased apoptosis after combination treatment in all three Doc-resistant cell lines. **C:** Microscopic images following the respective treatment after 3 days. Data are expressed as means ± SEM from at least three independent experiments. ∗*P* < 0.05; ∗∗*P* < 0.01; ∗∗∗*P* < 0.001. Scale bars = 200 μm. Original magnification, ×4.

Treatment with RU-486 combined with docetaxel using PC3-DR, DU145-DR, and CWR22Rv1-DR cell lines reduced cell viability. In contrast, this effect was absent when DR cell lines were treated individually with only docetaxel or RU-486 (Figure 1A). Prior studies indicated only a minor effect of RU-486 on parental PC3 and DU145 cells.^36^ Flow cytometry confirmed a significant increase in apoptosis, as indicated by an increased sub-G1 peak (Figure 1B). This increase was present in all three cell lines, independent of the concentrations used (3 μmol/L or 10 μmol/L of RU-486). However, this effect was more pronounced at higher RU-486 concentrations (Figure 1B). In concordance, macroscopic cell death in all three cell lines was observed after combination treatment (Figure 1C).

**Figure 2:**
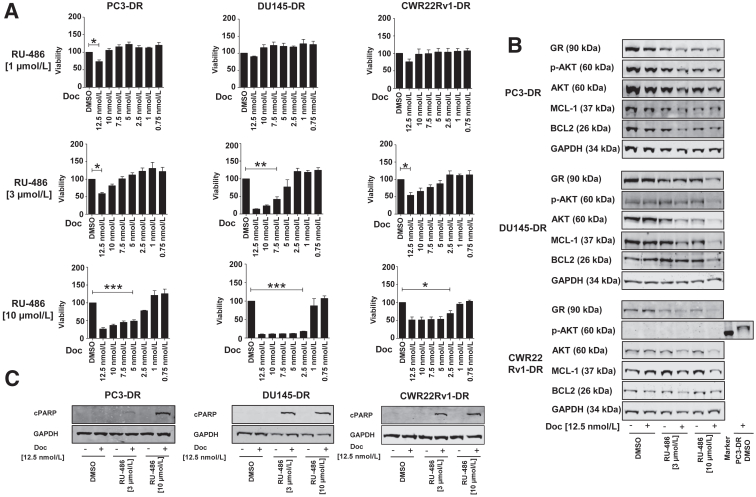
**A:** Measurement of cell viability after treatment with different concentrations of docetaxel (Doc) in all three cell lines. **B:** Western blot analysis of apoptotic and antiapoptotic cell cycle proteins in each cell line. **C:** Analysis of the apoptosis marker cPARP. All experiments have been performed at least three times. ∗*P* < 0.05; ∗∗*P* < 0.01; ∗∗∗*P* < 0.001. GAPDH, glyceraldehyde-3-phosphate dehydrogenase.

Next, the effect of different docetaxel concentrations on cell viability was assessed. For this purpose, all three cell lines were treated with increasing concentrations of docetaxel combined with increasing concentrations of RU-486 (1 μmol/L, 3 μmol/L, and 10 μmol/L). A significant reduction in viability was observed starting with docetaxel (2.5 nmol/L) together with 10 μmol/L RU-486 for DU145-DR and CWR22Rv1-DR, and 5 nmol/L docetaxel combined with 10 μmol/L RU-486 for PC3-DR (Figure 2A). Reducing the concentration of RU-486 to 3 μmol/L showed a similar significant reduction in viability but to a lesser extent. Reduction with 1 μmol/L RU-486 resulted in almost no change in cell viability (Figure 2A).

The expression of p-AKT, AKT, MCL-1, and BCL2, which are involved in cell proliferation and apoptosis, was assessed next. AKT phosphorylation and the general expression of selected antiapoptotic proteins were reduced compared with the controls in all three cell lines (Figure 2B). The AKT pathway was selected because of its central role in apoptosis, in which members of the Bcl-2 family play a role. Furthermore, the expression of GR was assessed, which was down-regulated in all three cell lines after combination treatment with docetaxel and RU-486 (Figure 2B). Notably, CWR22Rv-DR did not express p-AKT in multiple Western blot tests (Figure 2B). To further explore the effects of apoptosis on all three cell lines, the expression of cleaved poly-ADP-ribose polymerase (cPARP) was investigated as an additional marker of apoptosis. As expected, cPARP protein expression increased after the combination treatment with docetaxel and RU-486 (Figure 2C).

### RNA-Sequencing Identifies SREBP as a Significantly Down-regulated Gene in DR Cells

**Figure 3:**
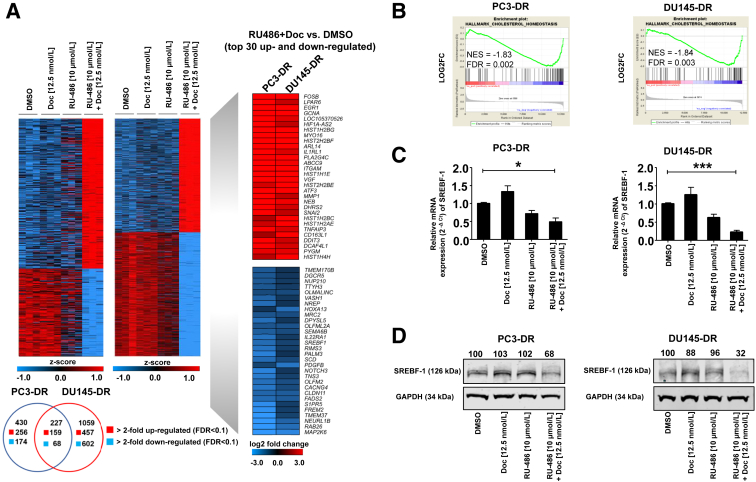
**A:** Results from RNA sequencing analyses [*z* score of log2 (normalized_counts+1)] represented as heatmaps after treatment with dimethyl sulfoxide (DMSO), docetaxel (Doc; 12.5 nmol/L), RU-486 (10 μmol/L), and the combination of Doc (12.5 nmol/L) and RU-486 (10 μmol/L) in PC3-DR and DU145-DR of significantly differentially expressed genes between RU-486 + Doc versus DMSO [more than twofold change; false discovery rate (FDR) <0.1] (**left**). Log2-fold changes of top 30 up- and down-regulated genes are indicated as heatmap (**right**). Venn diagram (**below**) showing overlap of significantly up-regulated and down-regulated genes of RU-486 + Doc versus DMSO treatment between PC3-DR and DU145-DR. **B:** Gene set enrichment analyses show a significantly elevated down-regulation of cholesterol homeostasis signature [normalized enrichment score (NES); FDR]. **C** and **D:** SREBF-1 was among the most significantly down-regulated genes with a significantly decreased mRNA expression in both cell lines (**C**) and significantly reduced protein levels in Western blot analysis confirming the results from the RNA sequencing data (**D**). ∗*P* < 0.05; ∗∗∗*P* < 0.001.

To identify the underlying mechanisms responsible for the observed apoptotic effects of combination treatment with docetaxel and RU-486, RNA-seq analysis was performed to identify altered gene expression profiles after single and combination treatment with docetaxel and RU-486 for 72 hours. RNA-seq revealed 227 overlapping genes in PC3-DR and DU145-DR cells, of which 159 genes were significantly up-regulated and 68 genes were significantly down-regulated after treatment with the combination of docetaxel and RU-486 (Figure 3A). Because an increase in apoptosis and cell death was observed under combination treatment, the focus was on genes that were significantly down-regulated. Subsequent gene set enrichment analysis identified cholesterol and lipid biosynthesis as significantly down-regulated pathways after combination treatment in both cell lines (Figure 3B). Among the 30 significantly down-regulated genes, *SREBF1* was chosen as a promising target gene for further experiments because SREBs are known to be associated with advanced PCa.^37^^,^^38^ To confirm the RNA-seq data, the protein and mRNA expression of SREBF-1 was examined in PC3-DR and DU145-DR cells after treatment with docetaxel, RU-486, or a combination of both. As expected, the relative mRNA expression and protein expression of SREBF-1 were significantly down-regulated in both cell lines under combination treatment (Figure 3, C and D). In summary, these results point to the potential involvement of SREBF-1 as a new factor for acquired therapy resistance and underline the hypothesis that elevated SREBF-1 expression plays an essential role in advanced PCa.^26^

### SREBF-1 mRNA Expression Is Significantly Increased in Primary PCa Tissue

**Figure 4:**
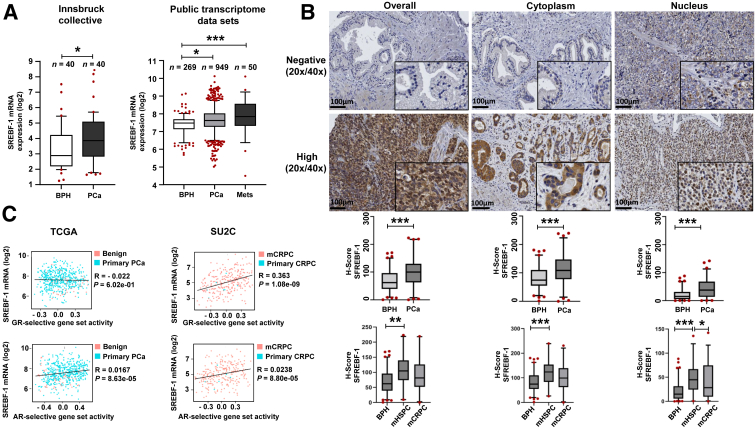
**A:** Significantly increased expression of sterol regulatory element of binding transcription factor 1 (SREBF-1) in 40 macro-dissected primary patients with prostate cancer (PCa) compared with tissue from 40 benign prostate samples as well as significantly increased expression of *SREBF1* of 949 patients with PCa and 50 PCa metastasis (Mets) samples compared with 269 benign tissue samples analyzed in public transcriptome data set. **B:** Representative microscopy images of negative and high expression of SREBF-1 in the overall cell compartment, the cytoplasm, and the nucleus as well as the corresponding statistical analysis between benign tissue and PCa tissue. In addition, patients with PCa were dichotomized into metastatic hormone-sensitive prostate cancer (mHSPC) and metastatic castration-resistant prostate cancer (mCRPC) groups in which SREBF-1 was more intensely expressed in mHSPC than mCRPC. However, this was not statistically significant. **C:** SREBF-1 significantly correlates with androgen receptor (AR) activity within the publicly available TCGA-PRAD (547 samples) data set. In the SU2C-PRAD data sets, AR and glucocorticoid (GR) activities were significantly positively correlated with *SREBF1*. Primary PCa is defined as mostly untreated PCa, whereas the SU2C database consists of heavily pretreated castration-resistant (CRPC) and metastatic PCa (mCRPC). **A:** unpaired *t*-test, ∗*P* < 0.05, ∗∗∗*P* < 0.001, box whisker plot with 10 to 90 percentile. **B:** ∗*P* < 0.05; ∗∗*P* < 0.01; ∗∗∗*P* < 0.001. Scale bars = 100 μm. Original magnification: ×20 (**main images**); ×40 (**insets**) (**B**). BPH, benign prostatic hyperplasia; TCGA, The Cancer Genome Atlas.

SREBF-1 mRNA expression was examined in 40 macro-dissected primary treatment-naive PCa specimens. SREBF-1 was significantly elevated in PCa samples compared with control samples (Figure 4A).

To confirm the RNA data obtained at the protein level, the expression of SREBF-1 in formalin-fixed paraffin-embedded tissue was assessed from transurethrally resected prostatic tissue samples from 72 patients with PCa who had progressive respective PCa and at least under androgen deprivation therapy due to biochemical recurrence (Table 2). SREBF-1 expression was significantly higher in PCa tissues than in benign tissues. To explore this observation in more detail, SREBF-1 expression was examined within the nucleus and cytoplasm of the cells as well as overall. Notably, SREBF-1 was expressed at significantly higher levels in all three cancer cell compartments than in the benign tissues (Figure 4B). Furthermore, because SREBF-1 is a transcription factor, its high expression in the nucleus can be directly correlated with elevated transcriptional activity.

**Table 2:** Patient Characteristics at TUR-P

Patient characteristics and descriptive histology at time of TUR-P	Overall population (*n* = 72)	mHSPC (*n* = 36)	mCRPC (*n* = 36)
	Means ± SD	Means ± SD	Means ± SD
Age at time of first TUR-P, y	*n* = 72	*n* = 36	*n* = 36
	75.3 ± 9.4	76 ± 10	74.7 ± 8
Survival from first TUR-P until time of death, months	*n* = 50	*n* = 25	*n* = 25
	23.3 ± 9.00	29.4 ± 27.3	17.1 ± 18
TUR-P resection volume (g)	*n* = 54	*n* = 28	*n* = 26
	17.47 ± 17.8	17.24 ± 21.7	17.71 ± 12.5
Statin medication			
Yes, % (yes/total)	23.6 (17/72)	30.6 (11/36)	16.7 (6/36)
No, % (no/total)	76.4 (55/72)	69.4 (25/36)	83.3 (30/36)
ISUP grade (Gleason score) in TUR-P specimen	n (%)	n (%)	n (%)
ISUP grade 1	3 (4.2)	3 (8.3)	0
ISUP grade 2 and 3	9 (12.5)	5 (13.9)	4 (11.1)
ISUP grade 4 and 5	39 (54.2)	18 (50.0)	21 (58.3)
Not defined	21 (29.2)	18 (50.0)	21 (58.3)

mCRPC, metastatic castration-resistant prostate cancer; mHSPC, metastatic hormone-sensitive prostate cancer; ISUP, International Society of Urological Pathology; TUR-P, transurethral palliative resection of the prostate.

Next, SREBF-1 expression in patients who were hormone-sensitive was compared with that in those who were castration-resistant at the time of transurethral resection of the prostate. SREBF-1 expression was significantly higher in tissues from patients with mHSPC and mCRPC than in benign tissues. However, no differences were found between mHSPC and mCRPC and respective SREBF-1 expression. To confirm this finding in an independent cohort, SREBF-1 mRNA expression was determined in an integrated cohort of four public transcriptome data sets [TCGA-PRAD, GSE21034, GSE35988, and GSE62872 (*https://www.ncbi.nlm.nih.gov/geo*)]. SREBF-1 was also expressed at significantly higher levels in PCa and metastatic PCa than in benign tissue. Notably, the highest SREBF-1 expression level was observed in metastatic PCa (Figure 4A). To assess the relationship between SREBF-1, GR, and AR, a correlation analysis was performed (Figure 4C) in publicly available PCa data sets from early treatment-naive PCa (TCGA-PRAD data set) and late-stage CRPC (SU2C data set). A very weak, albeit significant, correlation was observed between SREBF-1 mRNA expression and AR-selective target gene set activity in both data sets. In contrast, a highly significant correlation was observed between SREBF-1 mRNA expression and GR-selective target gene set activity that was specific to the late-stage CRPC data set.

### siRNA Knockdown of SREBF-1 Leads to Reduced Viability and Increased Apoptosis in DR Cell Lines

**Figure 5:**
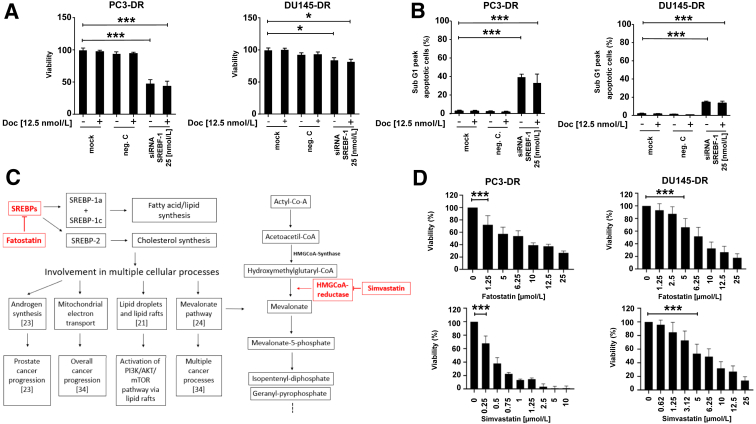
Confirmation of functional inhibition of the target gene SREBF-1 with siRNA knockdown in PC3-DR and DU145-DR. **A** and **B:** Viability assay (**A**) and flow cytometry analysis (**B**) show increased apoptosis after siRNA knockdown of the target gene SREBF-1. **C:** Diagram of sterol regulatory element binding proteins (SREBPs) and their involvement in the cellular processes of cancer as well as point of action for fatostatin and simvastatin. Simavastatin and fatostatin and their primary target of inhibition are marked in red. **D:** Viability assays with pharmacologic inhibition of fatostatin and simvastatin show decreased viability with increasing concentrations of fatostatin and simvastatin. ∗*P* < 0.05; ∗∗∗*P* < 0.001. CoA, coenzyme-A; HMGCoA, 3-hydroxy-3-methylglutaryl-coenzyme-A; mTOR, mammalian target of rapamycin; SREBF-1, sterol regulatory element of binding transcription factor 1.

To elucidate the functional effect on DR PCa after specific SREBF-1 down-regulation, transient siRNA knockdown was performed in PC3-DR and DU145-DR cells. After confirmation of down-regulation of SREBF-1 (Supplemental Figure S1, A and B), further analysis revealed significantly reduced cell viability and increased apoptosis after SREBF-1 knockdown (Figure 5, A and B). Decreased expression of SREBF-1 after treatment with RU-486 and docetaxel was also observed in AR-positive DR subline CWR22Rv1-DR cells (Supplemental Figure S1C).

### Pharmacologic Inhibition of SREBF-1 and Cholesterol Biosynthesis via Fatostatin and Simvastatin

To explore the functional relevance of pharmacologic SREB and cholesterol synthesis inhibition, further assays were performed with the SREBP inhibitor fatostatin. In addition, because fatostatin is not approved by the US Food and Drug Administration or the Emergency Medicines Agency, simvastatin was used for functional viability tests. Simvastatin is a highly active, US Food and Drug Administration– and Emergency Medicines Agency–approved inhibitor of HMGCoA-R, the first rate-limiting enzyme of the mevalonate pathway (Figure 5C). Viability tests showed a significant dose-dependent inhibitory effect of fatostatin and simvastatin on PC3-DR and DU145-DR (Figure 5D). SREBF1 expression was reduced in DR cells after treatment with fatostatin (Supplemental Figure S1D). These results therefore confirmed the relevance of both SREBs and HMGCoA-R in DR cell lines.

## Discussion

Elevated GR signaling is involved in PCa progression, and inhibition of GR signaling in combination with docetaxel results in reduced viability of DR PCa cells.^14^^,^^36^ Because docetaxel treatment primarily inhibits microtubular depolymerization in PCa cells and leads to G2/M cell arrest, the current results of increased apoptosis implicate a novel finding that was used as the basis to explore this observation further.^39^ To unravel the findings of increased apoptosis after combination treatment with docetaxel and the GR inhibitor RU-486, an RNA-seq approach was chosen to identify potential genes of interest that correlated with this observation. The cholesterol and lipid biosynthesis pathways were significantly altered. Specifically, SREBF-1 was among the most highly down-regulated genes after combination treatment with docetaxel and RU-486 in DU145-DR and PC3-DR cells. Increased expression of key enzymes in the mevalonate pathway, such as squalene epoxidase and SREBPs, has already been identified as playing a pivotal role in advanced PCa.^40^, ^41^, ^42^ However, the specific relevance of SREBPs in the setting of docetaxel resistance in PCa, as well as the expression of SREBPs in mHSPC compared with mCRPC, has not yet been investigated, and represents a novelty of this work.

In this context, high SREBP expression is associated with increased PCa cell survival, tumor aggressiveness, and progression to advanced PCa and other cancer types.^26^^,^^37^^,^^43^ Using an LnCaP-xenograft model, Ettinger et al^26^ showed that SREBPs are increasingly expressed with the emergence of androgen independence. These results concur with a report by Huang et al,^44^^,^^45^ who observed an association between increased SREBF-1 expression and tumor aggressiveness in AR-positive cell lines. Furthermore, SREBPs seem to regulate AR activity by inducing AR gene expression, which is a key driver of PCa progression and remains the prime target in modern PCa therapy.^46^, ^47^, ^48^ Likewise, overexpression of SREBP-1 increases cell proliferation, migration, and invasion.^45^ In a study by Nguyen et al,^49^ nuclear SREBF-1 expression was significantly increased in the late stages of PCa, supporting its involvement in advanced PCa.

Although we omitted the DR androgen-positive cell line CWR22Rv1-DR in our RNA-seq analysis, a similar apoptotic effect, reduced viability as well as decreased mRNA and protein expression after combination treatment, was observed. This finding implies an impact on survival in AR-positive cell lines after down-regulation of cholesterol and lipid biosynthesis. SREB knockdown also leads to reduced cell proliferation in docetaxel-naive CWR22Rv1 cells, although the research group did not use DR cell models compared with our experimental approach.^38^

These results support and further expand the idea that SREBPs are essential regulators of PCa aggressiveness and docetaxel resistance. First, the experimental approach was designed using a DR cell model. This result is novel compared with that of previous studies in which androgen-independent or docetaxel-naive cell lines were used. Therefore, lowering the activity of cholesterol and lipid biosynthesis pathways might be an overlapping point of attack against androgen independence and docetaxel resistance in future therapies.^50^ Second, the current data and analysis of public transcriptome data sets showed increased SREBF-1 mRNA expression in localized PCa, which was even more pronounced in metastatic PCa than in benign tissue, implicating an increasing relevance of SREBPs in the process of therapy resistance. Notably, there was a significant correlation between GR activity and elevated SREBF-1 expression in the late-stage CRPC SU2C data set, confirming the involvement of SREBs in aggressive PCa. In another study, Audet-Walsh et al^51^ showed that SREBF-1 is increasingly expressed under the AR/mammalian target of rapamycin axis, thereby implicating metabolic vulnerability.

To further translate these findings into a clinical setting, pharmacologic inhibition of SREBPs was performed with fatostatin and simvastatin, the latter of which is one of the most commonly prescribed HMGCoA-R inhibitors for lowering cholesterol levels against hyperlipidemia. Interestingly, pharmacologic inhibition with fatostatin and simvastatin was comparable to the initial findings after combination treatment with docetaxel and RU-486. This result aligns with the data from Li et al,^52^ who investigated the effects of SREBP inhibition by fatostatin using LNCaP and C4-2B PCa cell lines and a xenograft model. They observed increased apoptosis and G2/M cell cycle arrest after fatostatin treatment. In addition, combination treatment with fatostatin and docetaxel resulted in the most prominent decreases in viability, colony formation, and tumor volume. Notably, their experiments revealed that mutant p53s, which activate the SREBP pathway, are more susceptible to the combination of fatostatin and docetaxel, resulting in increased apoptosis and reduced proliferation. Kong et al^20^ investigated the additive efficacy of HMGCoA-R inhibition via simvastatin in combination with a second-generation antihormonal agent, enzalutamide. They observed elevated HMGCoA-R expression with the emergence of enzalutamide resistance, and combination treatment with enzalutamide and simvastatin re-induced enzalutamide sensitivity in enzalutamide-resistant MR49F PCa cells. The current pharmacologic experiments further expanded these findings by showing clear efficacy in DR cell models. These results provide a potential clinical approach for increasing the efficacy of treatment for highly advanced and metastatic PCa.

Finally, tissue specimen staining from highly selective patients with PCa provides several exciting insights. First, the *in vitro* results of increased SREBF-1 expression in PC3-DR and DU145-DR cell models were translated into the patient. SREBF-1 mRNA and protein levels were significantly higher in primary and metastatic PCa than in benign tissues. Therefore, these observations imply increased SREBF-1 expression at the mRNA level in *in vitro* models and increased protein activity in patient-derived materials, implicating translational and clinical relevance of this protein. This led to the second novel aspect: SREBF-1 expression was significantly higher in the total cell analysis, cytoplasm, and within the nucleus in immunohistochemical staining. After specific activation of SREBF-1 via steroid regulatory element-binding protein clearance activating protein (SCAP) at the NH2-terminal region, SREBF-1 translocates to the nucleus and acts as a transcription factor by regulating specific target genes from the cholesterol and lipid biosynthesis pathways.^53^ Notably, additional analysis of SREBF-1 staining in the nuclear compartment revealed generally higher SREBF-1 protein expression in the screened cancer tissues and confirmed the nuclear localization of this protein. These observations underline the relevance of SREBF-1 as an active enzyme in our cohort of patients with advanced PCa. Third, SREBPs such as fatostatin are vulnerable to statin therapy. Although fatostatin is not approved by the US Food and Drug Administration, further clinical trials are needed to reveal the true impact of statin inhibition of cholesterol and lipid biosynthesis on PCa therapy with fatostatin or other US Food and Drug Administration–approved therapies such as simvastatin.

Despite these promising results, the current study had some limitations. Our assumption of increased efficacy of statin inhibition in DR, AR-positive cell lines, such as CWR22Rv1, is based on the initial observation of increased apoptosis and reduced viability after combination treatment with docetaxel and mifepristone. These results were confirmed in CWR22Rv1-DR cells. Altered SREBF-1 mRNA and protein expression were in concordance with results gained in PC3-DR and DU145-DR cells. Therefore, targeting SREBF-1 also seems promising in androgen-positive DR cells. The subsequent experiments were not performed using this specific cell line. However, we hypothesized that specific cholesterol inhibition via SREBs would also have a significant impact on AR-positive cell lines. Future experiments in this direction are of great interest. Second, the efficacy of combination therapy with docetaxel and mifepristone in *in vivo* models remains debatable. Yang et al^54^ used C4-2 cells for an *in vivo* experiment, and their results showed limited activity of combination therapy with docetaxel and mifepristone. Thus, the question remains open whether this therapy improves docetaxel efficacy, but at this time, we have no evidence of it. However, it must be mentioned that their approach was not used in a DR tumor cell model, which might be a reason for the decreased efficacy. Furthermore, it must be mentioned that there is also a clinical phase 1/2 study that combined enzalutamide and mifepristone, which also did not result in increased efficacy of enzalutamide.^55^ Lastly, the interaction between GR signaling and SREBPs was not further evaluated in this study; this interaction would also be of great interest.

In conclusion, this study identified SREBF-1 as an essential regulator of cell survival and susceptibility to docetaxel in advanced and metastatic PCa. These data outline the strong involvement of SREBPs in metastatic PCa and provide further evidence that inhibiting cholesterol and lipid biosynthesis might provide a clinically meaningful rationale for increasing the efficacy of PCa therapies and in specific DR PCa.

## Supplemental Data

Supplemental Figure S1 **A** and **B:** Relative mRNA expression of sterol regulatory element of binding transcription factor 1 (SREBF-1) after siRNA knockdown in PC3-DR cells after treatment with 25 nmol/L and 50 nmol/L siRNA SMARTpool for SREBF-1 (**A**) as well as Western blot analysis (**B**). **C:** Relative mRNA of SREBF-1 and Western blot analysis of SREBF-1 in the androgen-positive cell line CWR22Rv1-DR after specific treatment shows significantly decreased mRNA expression with significantly reduced protein levels in Western blot analysis. **D:** Confirmation with immunohistochemical staining of SREBF-1 in PC3-DR and DU145-DR after treatment with fatostatin. For this purpose, the cells were prepared from cell culture and harvested with Accutase (Innovative Cell Technologies, San Diego, CA). After centrifugation at 300 × *g* at 4°C for 5 minutes, cells were washed in phosphate-buffered saline and fixed in 4% formalin at 37°C for 15 minutes. After another wash step with phosphate-buffered saline, the cells were transferred to 100% ethanol and precipitated with 30% fetal calf serum. For immunostaining, cell pellets were subsequently fixed in formalin, embedded in paraffin and sectioned into 2 μm slices according to standard protocols. ∗∗*P* < 0.01. Scale bars = 100 μm. DMSO, dimethyl sulfoxide.

## Footnotes

Supplemental material for this article can be found at *http://doi.org/10.1016/j.ajpath.2024.07.019*.

## References

[1] Smith M.R., Saad F., Chowdhury S., Oudard S., Hadaschik B.A., Graff J.N., Olmos D., Mainwaring P.N., Lee J.Y., Uemura H., Lopez-Gitlitz A., Trudel G.C., Espina B.M., Shu Y., Park Y.C., Rackoff W.R., Yu M.K., Small E.J. (2018). Apalutamide treatment and metastasis-free survival in prostate cancer. N Engl J Med.

[2] Fizazi K., Scher H.I., Molina A., Logothetis C.J., Chi K.N., Jones R.J., Staffurth J.N., North S., Vogelzang N.J., Saad F., Mainwaring P., Harland S., Goodman O.B., Sternberg C.N., Li J.H., Kheoh T., Haqq C.M., de Bono J.S., COU-AA-301 Investigators (2012). Abiraterone acetate for treatment of metastatic castration-resistant prostate cancer: final overall survival analysis of the COU-AA-301 randomised, double-blind, placebo-controlled phase 3 study. Lancet Oncol.

[3] Fizazi K., Shore N., Tammela T.L., Ulys A., Vjaters E., Polyakov S., Jievaltas M., Luz M., Alekseev B., Kuss I., Kappeler C., Snapir A., Sarapohja T., Smith M.R., ARAMIS Investigators (2019). Darolutamide in nonmetastatic, castration-resistant prostate cancer. N Engl J Med.

[4] James N.D., de Bono J.S., Spears M.R., Clarke N.W., Mason M.D., Dearnaley D.P. (2017). Abiraterone for prostate cancer not previously treated with hormone therapy. N Engl J Med.

[5] James N.D., Sydes M.R., Clarke N.W., Mason M.D., Dearnaley D.P., Spears M.R. (2016). Addition of docetaxel, zoledronic acid, or both to first-line long-term hormone therapy in prostate cancer (STAMPEDE): survival results from an adaptive, multiarm, multistage, platform randomised controlled trial. Lancet.

[6] Beer T.M., Armstrong A.J., Rathkopf D.E., Loriot Y., Sternberg C.N., Higano C.S., Iversen P., Bhattacharya S., Carles J., Chowdhury S., Davis I.D., de Bono J.S., Evans C.P., Fizazi K., Joshua A.M., Kim C.S., Kimura G., Mainwaring P., Mansbach H., Miller K., Noonberg S.B., Perabo F., Phung D., Saad F., Scher H.I., Taplin M.E., Venner P.M., Tombal B., Investigators P. (2014). Enzalutamide in metastatic prostate cancer before chemotherapy. N Engl J Med.

[7] Sweeney C.J., Chen Y.-H., Carducci M., Liu G., Jarrard D.F., Eisenberger M., Wong Y.-N., Hahn N., Kohli M., Cooney M.M., Dreicer R., Vogelzang N.J., Picus J., Shevrin D., Hussain M., Garcia J.A., DiPaola R.S. (2015). Chemohormonal therapy in metastatic hormone-sensitive prostate cancer. N Engl J Med.

[8] Tannock I.F., de Wit R., Berry W.R., Horti J., Pluzanska A., Chi K.N., Oudard S., Théodore C., James N.D., Turesson I., Rosenthal M.A., Eisenberger M.A., 327 Investigators T.A.X. (2004). Docetaxel plus prednisone or mitoxantrone plus prednisone for advanced prostate cancer. N Engl J Med.

[9] Smith M.R., Hussain M., Saad F., Fizazi K., Sternberg C.N., Crawford E.D., Kopyltsov E., Park C.H., Alekseev B., Montesa-Pino A., Ye D., Parnis F., Cruz F., Tammela T.L.J., Suzuki H., Utriainen T., Fu C., Uemura M., Méndez-Vidal M.J., Maughan B.L., Joensuu H., Thiele S., Li R., Kuss I., Tombal B., Investigators (2022). Darolutamide and survival in metastatic, hormone-sensitive prostate cancer. N Engl J Med.

[10] Fizazi K., Foulon S., Carles J., Roubaud G., McDermott R., Flechon A., Tombal B., Supiot S., Berthold D., Ronchin P., Kacso G., Gravis G., Calabro F., Berdah J.F., Hasbini A., Silva M., Thiery-Vuillemin A., Latorzeff I., Mourey L., Laguerre B., Abadie-Lacourtoisie S., Martin E., El Kouri C., Escande A., Rosello A., Magne N., Schlurmann F., Priou F., Chand-Fouche M.E., Freixa S.V., Jamaluddin M., Rieger I., Bossi A., Investigators P- (2022). Abiraterone plus prednisone added to androgen deprivation therapy and docetaxel in de novo metastatic castration-sensitive prostate cancer (PEACE-1): a multicentre, open-label, randomised, phase 3 study with a 2 × 2 factorial design. Lancet.

[11] Yehya A., Ghamlouche F., Zahwe A., Zeid Y., Wakimian K., Mukherji D., Abou-Kheir W. (2022). Drug resistance in metastatic castration-resistant prostate cancer: an update on the status quo. Cancer Drug Resist.

[12] Rizzo M. (2021). Mechanisms of docetaxel resistance in prostate cancer: the key role played by miRNAs. Biochim Biophys Acta Rev Cancer.

[13] Seruga B., Ocana A., Tannock I.F. (2011). Drug resistance in metastatic castration-resistant prostate cancer. Nat Rev Clin Oncol.

[14] Puhr M., Hoefer J., Eigentler A., Ploner C., Handle F., Schaefer G., Kroon J., Leo A., Heidegger I., Eder I., Culig Z., Van der Pluijm G., Klocker H. (2018). The glucocorticoid receptor is a key player for prostate cancer cell survival and a target for improved antiandrogen therapy. Clin Cancer Res.

[15] Arora V.K., Schenkein E., Murali R., Subudhi S.K., Wongvipat J., Balbas M.D., Shah N., Cai L., Efstathiou E., Logothetis C., Zheng D., Sawyers C.L. (2013). Glucocorticoid receptor confers resistance to antiandrogens by bypassing androgen receptor blockade. Cell.

[16] Rane J.K., Erb H.H.H., Nappo G., Mann V.M., Simms M.S., Collins A.T., Visakorpi T., Maitland N.J. (2016). Inhibition of the glucocorticoid receptor results in an enhanced miR-99a/100-mediated radiation response in stem-like cells from human prostate cancers. Oncotarget.

[17] Leon C.G., Locke J.A., Adomat H.H., Etinger S.L., Twiddy A.L., Neumann R.D., Nelson C.C., Guns E.S., Wasan K.M. (2010). Alterations in cholesterol regulation contribute to the production of intratumoral androgens during progression to castration-resistant prostate cancer in a mouse xenograft model. Prostate.

[18] Beier A.-M.K., Puhr M., Stope M.B., Thomas C., Erb H.H.H. (2023). Metabolic changes during prostate cancer development and progression. J Cancer Res Clin Oncol.

[19] Rehman Y., Rosenberg J.E. (2012). Abiraterone acetate: oral androgen biosynthesis inhibitor for treatment of castration-resistant prostate cancer. Drug Des Devel Ther.

[20] Kong Y., Cheng L., Mao F., Zhang Z., Zhang Y., Farah E., Bosler J., Bai Y., Ahmad N., Kuang S., Li L., Liu X. (2018). Inhibition of cholesterol biosynthesis overcomes enzalutamide resistance in castration-resistant prostate cancer (CRPC). J Biol Chem.

[21] Zhang G.-C., Yu X.-N., Guo H.-Y., Sun J.-L., Liu Z.-Y., Zhu J.-M., Liu T.-T., Dong L., Shen X.-Z., Yin J. (2023). PRP19 enhances esophageal squamous cell carcinoma progression by reprogramming SREBF1-dependent fatty acid metabolism. Cancer Res.

[22] Lewis C.A., Brault C., Peck B., Bensaad K., Griffiths B., Mitter R., Chakravarty P., East P., Dankworth B., Alibhai D., Harris A.L., Schulze A. (2015). SREBP maintains lipid biosynthesis and viability of cancer cells under lipid- and oxygen-deprived conditions and defines a gene signature associated with poor survival in glioblastoma multiforme. Oncogene.

[23] Bao J., Zhu L., Zhu Q., Su J., Liu M., Huang W. (2016). SREBP-1 is an independent prognostic marker and promotes invasion and migration in breast cancer. Oncol Lett.

[24] Vona R., Iessi E., Matarrese P. (2021). Role of cholesterol and lipid rafts in cancer signaling: a promising therapeutic opportunity?. Front Cell Dev Biol.

[25] Eberhard Y., Gronda M., Hurren R., Datti A., MacLean N., Ketela T., Moffat J., Wrana J.L., Schimmer A.D. (2011). Inhibition of SREBP1 sensitizes cells to death ligands. Oncotarget.

[26] Ettinger S.L., Sobel R., Whitmore T.G., Akbari M., Bradley D.R., Gleave M.E., Nelson C.C. (2004). Dysregulation of sterol response element-binding proteins and downstream effectors in prostate cancer during progression to androgen independence. Cancer Res.

[27] Shao W., Espenshade P.J. (2012). Expanding roles for SREBP in metabolism. Cell Metab.

[28] Puhr M., Hoefer J., Schäfer G., Erb H.H.H., Oh S.J., Klocker H., Heidegger I., Neuwirt H., Culig Z. (2012). Epithelial-to-mesenchymal transition leads to docetaxel resistance in prostate cancer and is mediated by reduced expression of miR-200c and miR-205. Am J Pathol.

[29] Puhr M., Santer F.R., Neuwirt H., Susani M., Nemeth J.A., Hobisch A., Kenner L., Culig Z. (2009). Down-regulation of suppressor of cytokine signaling-3 causes prostate cancer cell death through activation of the extrinsic and intrinsic apoptosis pathways. Cancer Res.

[30] Liu J., Lichtenberg T., Hoadley K.A., Poisson L.M., Lazar A.J., Cherniack A.D., Kovatich A.J., Benz C.C., Levine D.A., Lee A.V., Omberg L., Wolf D.M., Shriver C.D., Thorsson V., Cancer Genome Atlas Research Network, Hu H. (2018). An integrated TCGA pan-cancer clinical data resource to drive high-quality survival outcome analytics. Cell.

[31] Taylor B.S., Schultz N., Hieronymus H., Gopalan A., Xiao Y., Carver B.S., Arora V.K., Kaushik P., Cerami E., Reva B., Antipin Y., Mitsiades N., Landers T., Dolgalev I., Major J.E., Wilson M., Socci N.D., Lash A.E., Heguy A., Eastham J.A., Scher H.I., Reuter V.E., Scardino P.T., Sander C., Sawyers C.L., Gerald W.L. (2010). Integrative genomic profiling of human prostate cancer. Cancer Cell.

[32] Grasso C.S., Wu Y.-M., Robinson D.R., Cao X., Dhanasekaran S.M., Khan A.P., Quist M.J., Jing X., Lonigro R.J., Brenner J.C., Asangani I.A., Ateeq B., Chun S.Y., Siddiqui J., Sam L., Anstett M., Mehra R., Prensner J.R., Palanisamy N., Ryslik G.A., Vandin F., Raphael B.J., Kunju L.P., Rhodes D.R., Pienta K.J., Chinnaiyan A.M., Tomlins S.A. (2012). The mutational landscape of lethal castration-resistant prostate cancer. Nature.

[33] Penney K.L., Sinnott J.A., Tyekucheva S., Gerke T., Shui I.M., Kraft P., Sesso H.D., Freedman M.L., Loda M., Mucci L.A., Stampfer M.J. (2015). Association of prostate cancer risk variants with gene expression in normal and tumor tissue. Cancer Epidemiol Biomarkers Prev.

[34] Abida W., Cyrta J., Heller G., Prandi D., Armenia J., Coleman I. (2019). Genomic correlates of clinical outcome in advanced prostate cancer. Proc Natl Acad Sci U S A.

[35] Bankhead P., Loughrey M.B., Fernández J.A., Dombrowski Y., McArt D.G., Dunne P.D., McQuaid S., Gray R.T., Murray L.J., Coleman H.G., James J.A., Salto-Tellez M., Hamilton P.W. (2017). QuPath: open source software for digital pathology image analysis. Sci Rep.

[36] Kroon J., Puhr M., Buijs J.T., van der Horst G., Hemmer D.M., Marijt K.A., Hwang M.S., Masood M., Grimm S., Storm G., Metselaar J.M., Meijer O.C., Culig Z., van der Pluijm G. (2016). Glucocorticoid receptor antagonism reverts docetaxel resistance in human prostate cancer. Endocr Relat Cancer.

[37] Griffiths B., Lewis C.A., Bensaad K., Ros S., Zhang Q., Ferber E.C., Konisti S., Peck B., Miess H., East P., Wakelam M., Harris A.L., Schulze A. (2013). Sterol regulatory element binding protein-dependent regulation of lipid synthesis supports cell survival and tumor growth. Cancer Metab.

[38] Li X., Wu J.B., Li Q., Shigemura K., Chung L.W.K., Huang W.-C. (2016). SREBP-2 promotes stem cell-like properties and metastasis by transcriptional activation of c-Myc in prostate cancer. Oncotarget.

[39] Pienta K.J. (2001). Preclinical mechanisms of action of docetaxel and docetaxel combinations in prostate cancer. Semin Oncol.

[40] Stopsack K.H., Gerke T.A., Andrén O., Andersson S.-O., Giovannucci E.L., Mucci L.A., Rider J.R. (2017). Cholesterol uptake and regulation in high-grade and lethal prostate cancers. Carcinogenesis.

[41] Raftopulos N.L., Washaya T.C., Niederprum A., Egert A., Hakeem-Sanni M.F., Varney B., Aishah A., Georgieva M.L., Olsson E., Dos Santos D.Z., Nassar Z.D., Cochran B.J., Nagarajan S.R., Kakani M.S., Hastings J.F., Croucher D.R., Rye K.-A., Butler L.M., Grewal T., Hoy A.J. (2022). Prostate cancer cell proliferation is influenced by LDL-cholesterol availability and cholesteryl ester turnover. Cancer Metab.

[42] Kalogirou C., Linxweiler J., Schmucker P., Snaebjornsson M.T., Schmitz W., Wach S., Krebs M., Hartmann E., Puhr M., Müller A., Spahn M., Seitz A.K., Frank T., Marouf H., Büchel G., Eckstein M., Kübler H., Eilers M., Saar M., Junker K., Rohrig F., Kneitz B., Rosenfeldt M.T., Schulze A. (2021). MiR-205-driven downregulation of cholesterol biosynthesis through SQLE-inhibition identifies therapeutic vulnerability in aggressive prostate cancer. Nat Commun.

[43] Jiang T., Zhang G., Lou Z. (2020). Role of the sterol regulatory element binding protein pathway in tumorigenesis. Front Oncol.

[44] Huang W.-C., Zhau H.E., Chung L.W.K. (2010). Androgen receptor survival signaling is blocked by anti-beta2-microglobulin monoclonal antibody via a MAPK/lipogenic pathway in human prostate cancer cells. J Biol Chem.

[45] Huang W.-C., Li X., Liu J., Lin J., Chung L.W.K. (2012). Activation of androgen receptor, lipogenesis, and oxidative stress converged by SREBP-1 is responsible for regulating growth and progression of prostate cancer cells. Mol Cancer Res.

[46] Erb H.H.H., Oster M.A., Gelbrich N., Cammann C., Thomas C., Mustea A., Stope M.B. (2021). Enzalutamide-induced proteolytic degradation of the androgen receptor in prostate cancer cells is mediated only to a limited extent by the proteasome system. Anticancer Res.

[47] Siciliano T., Simons I.H., Beier A.-M.K., Ebersbach C., Aksoy C., Seed R.I., Stope M.B., Thomas C., Erb H.H.H. (2021). A systematic comparison of antiandrogens identifies androgen receptor protein stability as an indicator for treatment response. Life (Basel).

[48] Siciliano T., Sommer U., Beier A.-M.K., Stope M.B., Borkowetz A., Thomas C., Erb H.H.H. (2022). The androgen hormone-induced increase in androgen receptor protein expression is caused by the autoinduction of the androgen receptor translational activity. Curr Issues Mol Biol.

[49] Nguyen T., Sridaran D., Chouhan S., Weimholt C., Wilson A., Luo J., Li T., Koomen J., Fang B., Putluri N., Sreekumar A., Feng F.Y., Mahajan K., Mahajan N.P. (2023). Histone H2A Lys130 acetylation epigenetically regulates androgen production in prostate cancer. Nat Commun.

[50] kara L., Huđek Turkovic A., Pezelj I., Vrtarić A., Sinčić N., Krušlin B., Ulamec M. (2021). Prostate cancer-focus on cholesterol. Cancers (Basel).

[51] Audet-Walsh E., Vernier M., Yee T., Laflamme C., Li S., Chen Y., Giguère V. (2018). SREBF1 activity is regulated by an AR/mTOR nuclear axis in prostate cancer. Mol Cancer Res.

[52] Li X., Wu J.B., Chung L.W.K., Huang W.-C. (2015). Anti-cancer efficacy of SREBP inhibitor, alone or in combination with docetaxel, in prostate cancer harboring p53 mutations. Oncotarget.

[53] Lee S.H., Lee J.-H., Im S.-S. (2020). The cellular function of SCAP in metabolic signaling. Exp Mol Med.

[54] Yang Y., Li X., Mamouni K., Kucuk O., Wu D. (2017). Mifepristone has limited activity to enhance the in vivo efficacy of docetaxel and enzalutamide against bone metastatic and castration-resistant prostate cancer. Anticancer Res.

[55] Serritella A.V., Shevrin D., Heath E.I., Wade J.L., Martinez E., Anderson A., Schonhoft J., Chu Y.-L., Karrison T., Stadler W.M., Szmulewitz R.Z. (2022). Phase I/II trial of enzalutamide and mifepristone, a glucocorticoid receptor antagonist, for metastatic castration-resistant prostate cancer. Clin Cancer Res.

