# Supplementary figures and images for: Inhibition of the Sterol Regulatory Element Binding Protein SREBF-1 Overcomes Docetaxel Resistance in Advanced Prostate Cancer
Source: Am J Pathol. 2024 Aug 19;194(11):2150–62. doi: 10.1016/j.ajpath.2024.07.019 (PMC12179511; doi:10.1016/j.ajpath.2024.07.019)

**A**

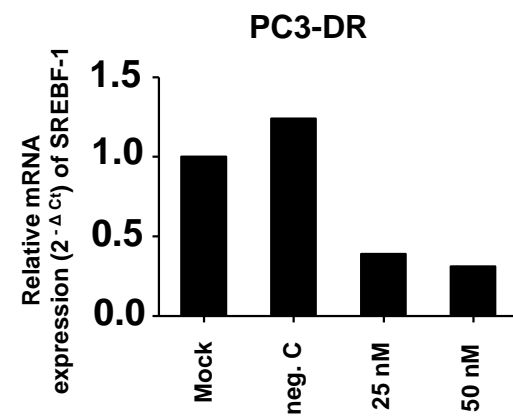

Negative (20x)

+ 10  $\mu$ M fatostatin

**B**

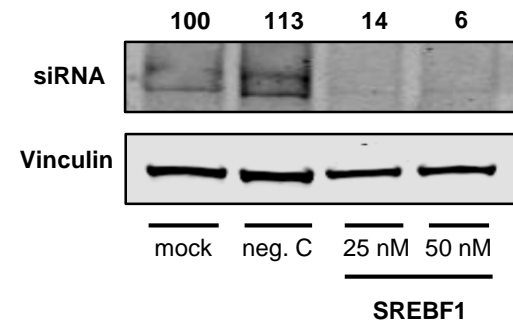

Positive (20x)

**C**

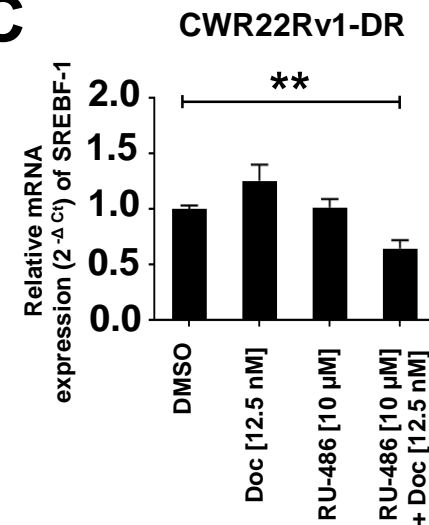

SREBF-1 (126kD)

GAPDH (34 kD)

**CWR22Rv1-DR**

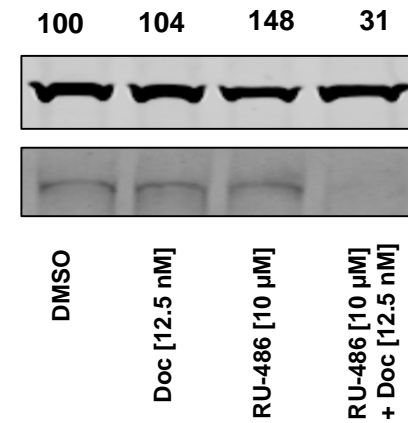

**D**

PC3-DR

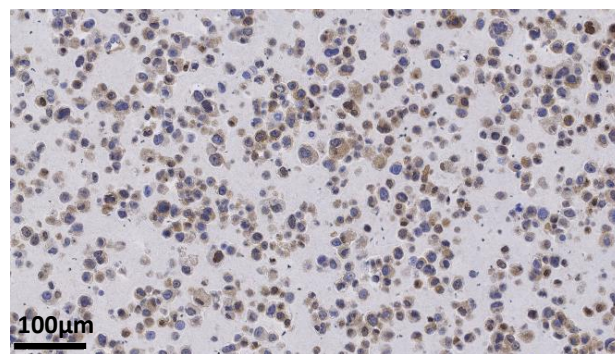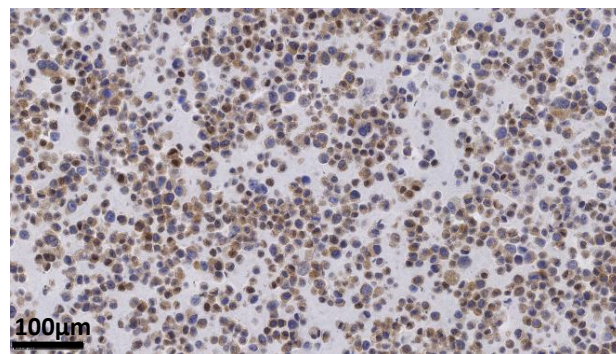

+ 6.25  $\mu$ M fatostatin

DU145-DR

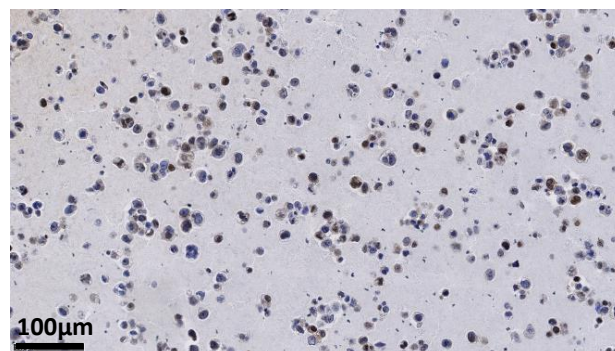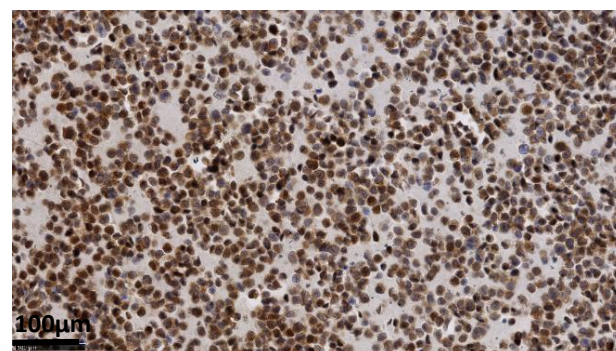

Supplement: Supplemental Figure S1 — A and B: Relative mRNA expression of sterol regulatory element of binding transcription factor 1 (SREBF-1) after siRNA knockdown in PC3-DR cells after treatment with 25 nmol/L and 50 nmol/L siRNA SMARTpool for SREBF-1 (A) as well as Western blot analysis (B). C: Relative mRNA of SREBF-1 and Western blot analysis of SREBF-1 in the androgen-positive cell line CWR22Rv1-DR after specific treatment shows significantly decreased mRNA expression with significantly reduced protein levels in Western blot analysis. D: Confirmation with immunohistochemical staining of SREBF-1 in PC3-DR and DU145-DR after treatment with fatostatin. For this purpose, the cells were prepared from cell culture and harvested with Accutase (Innovative Cell Technologies, San Diego, CA). After centrifugation at 300 × g at 4°C for 5 minutes, cells were washed in phosphate-buffered saline and fixed in 4% formalin at 37°C for 15 minutes. After another wash step with phosphate-buffered saline, the cells were transferred to 100% ethanol and precipitated with 30% fetal calf serum. For immunostaining, cell pellets were subsequently fixed in formalin, embedded in paraffin and sectioned into 2 μm slices according to standard protocols. ∗∗P < 0.01. Scale bars = 100 μm. DMSO, dimethyl sulfoxide. [file mmc1.pdf]
